# Flexibility of the Cytoplasmic Domain of the Phototaxis Transducer II from *Natronomonas pharaonis*

**DOI:** 10.1155/2008/267912

**Published:** 2008-10-16

**Authors:** Ivan L. Budyak, Olga S. Mironova, Naveena Yanamala, Vijayalaxmi Manoharan, Georg Büldt, Ramona Schlesinger, Judith Klein-Seetharaman

**Affiliations:** ^1^Institute for Structural Biology (IBI-2), Research Center Jülich, 52425 Jülich, Germany; ^2^Department of Structural Biology, University of Pittsburgh School of Medicine, Pittsburgh, PA 15260, USA

## Abstract

Chemo- and phototaxis systems in bacteria and archaea serve as models for more complex signal transduction mechanisms in higher eukaryotes. Previous studies of the cytoplasmic fragment of the phototaxis transducer (pHtrII-cyt) from the halophilic archaeon *Natronomonas pharaonis* showed that it takes the shape of a monomeric or dimeric rod under low or high salt conditions, respectively. CD spectra revealed only approximately 24% helical structure, even in 4 M KCl, leaving it an open question how the rod-like shape is achieved. Here, we conducted CD, FTIR, and NMR spectroscopic studies under different conditions to address this question. We provide evidence that pHtrII-cyt is highly dynamic with strong helical propensity, which allows it to change from monomeric to dimeric helical coiled-coil states without undergoing dramatic shape changes. A statistical analysis of predicted disorder for homologous sequences suggests that structural flexibility is evolutionarily conserved within the methyl-accepting chemotaxis protein family.

## 1. Introduction

Response to external stimuli is important for all living organisms. Prokaryotes and lower eukaryotes are able to move towards favorable and away from unfavorable conditions including chemicals and light, referred to as chemotaxis and phototaxis, respectively [1, 2]. Repellent phototaxis in the halophilic archaeon *Natronomonas pharaonis* (also known as *Natronobacterium pharaonis*) is initiated by light activation of sensory rhodopsin II (pSRII), a 7 *α*-helical membrane receptor related to bacteriorhodopsin [3]. Activation of pSRII induces structural changes in its cognate transducer protein, pHtrII, a 2 *α*-helical membrane protein with a long cytoplasmic extension that transmits the signal further to induce subsequent phosphorylation processes that control flagellar motion [2]. The crystal structures of pSRII alone [4, 5] and in complex with the transmembrane domain of pHtrII (residues 1–114, electron density was observed for residues 22–82) have been recently solved, both in the inactive dark state and in the light-activated conformation [6, 7]. A peptide fragment corresponding to the juxtamembrane residues 83–149 of the above protein was shown by NMR spectroscopy to be unstructured in solution, but to undergo a structural transition upon interaction with pSRII [8]. EPR spectroscopy of the N-terminal pHtrII protein fragment (residues 1–157) also showed that the juxtamembrane domain has molten globule-like characteristics [9]. However, the structure of the long cytoplasmic domain of pHtrII (pHtrII-cyt), which directly interacts with the proteins of the signaling cascade, remains unknown.

pHtrII-cyt exhibits sequence homology to the cytoplasmic domains of receptors in eubacteria including the well-studied *Escherichia coli* serine (Tsr-cyt) and aspartate (Tar-cyt) receptors [10]. The identity between pHtrII-cyt and the corresponding sequence of Tsr-cyt is 24%. For comparison, the overall identity between Tsr-cyt and Tar-cyt is 71%. These sequences belong to the methyl-accepting chemotaxis protein (MCP) family, which currently (as of March 2008) has more than 5000 identified members [11]. The functional significance of the homology between phototaxis transducer proteins and chemoreceptors has been proven through studies of chimera [12].

Tsr-cyt has been crystallized and its crystal structure corresponds to a helical coiled-coil dimer [13]. A comparison of the crystal structure of the methylated with the structure of the non-methylated signaling state revealed little changes in the structure, but significant differences in B-factor values [14] supporting a model, in which receptor activity is determined by the flexibility of the cytoplasmic domain [15]. Furthermore, the helical Tar-cyt fragment is easily denatured thermally [16, 17] and has the characteristics of a molten globule [18]. NMR amide-exchange data revealed the presence of a small (10%) core of slowly exchanging residues, which presumably are involved in tertiary packing contacts [18]. The size of the core increased as a result of mutation or pH change [16, 17, 19].

We have previously shown that pHtrII-cyt assumes a shape consistent with the Tsr-cyt helical coiled-coil dimer structure, but CD spectra lacked evidence for a predominantly helical conformation [20]. Here, we present an in-depth analysis of pHtrII-cyt using CD, Fourier-transform IR (FTIR), NMR spectroscopy, and statistical sequence analysis. From these studies, a picture emerges in which pHtrII-cyt has a high propensity for helical coiled-coil structure, but maintains flexibility to such an extent that CD is not able to reliably detect it.

## 2. Materials and Methods

### 2.1. Materials

All sources of materials including preparation of pHtrII-cyt were as described previously [20], unless otherwise stated. Buffers used were phosphate-buffered saline, 0.9 mM CaCl_2_, 0.5 mM MgCl_2_, 2.7 mM KCl, 138 mM NaCl, 1.5 mM KH_2_PO_4_, 8 mM Na_2_HPO_4_ (PBS); 10 mM NaP_i_ pH 6.0 (buffer A); 10 mM Tris-HCl pH 8.0 (buffer B); 10 mM Tris-HCl pH 8.0 in 99% D_2_O (buffer C); 10 mM NaP_i_ pH 6.0, 10% D_2_O (buffer D).

### 2.2. CD Spectroscopy

CD spectra were acquired using a Jasco 810 spectropolarimeter (Jasco Inc., Easton, Md, USA) as described previously [20]. Experimental data *θ*_exp_ were recorded in a 0.2-cm quartz cell at pHtrII-cyt concentration of 0.1 mg/mL and then converted to the mean residue ellipticity according to [*θ*]_MRW_ = *θ*_exp _/(10*l*c*n) where *l* is the path length of the cell in cm, *c* is the protein concentration in M, and *n* is the number of residues. [*θ*]_MRW_ was used as the input data for the CONTIN/LL secondary structure prediction program from the CDPro software package [21, 22]. The dataset including reference spectra of denaturated proteins (SDP48) was chosen as the reference one [23]. The error bars represent the standard deviation of values predicted by CONTIN/LL assuming 10% error in the determination of concentration. The values obtained from deconvolution should be considered approximate because of inherent difficulties in CD spectra deconvolution [24, 25] and the known effects of dynamics on reliability of secondary structure prediction from CD data [26]. However, we use them here to allow quantitative comparison of structure and dynamics of pHtrII-cyt under different solvent and temperature conditions.

### 2.3. NMR Spectroscopy

Measurements were performed at +20°C unless stated otherwise. Solutions for NMR spectroscopic analysis contained 5 mg/mL uniformly ^15^N-labeled pHtrII-cyt in buffer D. ^1^H-^15^N-HSQC, longitudinal (*T*_1_) and transverse (*T*_2_) relaxation data, and ^1^H-^15^N heteronuclear NOE (hetNOE) data of pHtrII-cyt in the absence and presence of 4 M salt were recorded at +10°C and +20°C using a Bruker 600 MHz spectrophotometer. Standard Bruker sequences were used to record ^1^H-, ^1^H-^15^N-HSQC, and het-NOE spectra. The *T*_1_, *T*_2_ spectra were recorded using the pulse sequence schemes described previously [27]. We used a 90° pulse of 50 microseconds for ^15^N for all the experiments and a 90° pulse of 14 microseconds and 9.6 microseconds for the ^1^H in 4 M salt and water samples, respectively. The *R*_1_(1/*T*_1_) and *R*_2_(1/*T*_2_) rates of the 4 M salt sample were measured using the relaxation delays of 40, 80, 160, 320, 480, 640 milliseconds and 30, 64, 96, 128, 192, 240 milliseconds, respectively. Further, the water relaxation rate values were obtained at relaxation delays of 32, 64, 128, 256, 384, 512 milliseconds for *R*_1_ and 32, 64, 128, 192, 256, 320 milliseconds for the *R*_2_. HetNOE experiments were obtained by collecting the spectra with and without ^1^H presaturation. A proton presaturation period of 3 seconds was applied before acquiring each ^1^H‐^15^N HSQC spectra. The data recorded were processed using the NMRPipe, NMRview, and Sparky software packages for further analysis. The *R*_1_ and *R*_2_ rates were estimated by fitting the peak intensities at different relaxation delays using the two-parameter exponential decay function in the numerical recipes package [27, 28]. The hetNOE values for each peak were estimated by finding the ratios between the peak intensities of the ^1^H-^15^N HSQC spectra with and without proton presaturation [27].

### 2.4. FTIR Spectroscopy

FTIR absorbance spectra were acquired at room temperature using a Bruker Vector 22 IR-spectrometer equipped with a DTGS detector. For film preparation, 150 *μ*g of pHtrII-cyt in buffer B were dried under N_2_ gas flow and then sealed. For measurements in solution, pHtrII-cyt was concentrated to 10 mg/mL, and buffer B was exchanged to buffer C to reduce background absorbance in the amide I region. 256 scans were taken for each sample at 1 cm^−1^ resolution. The spectrum of residual water vapor was measured with an empty chamber and then subtracted manually from the protein spectra.

### 2.5. Sequence-Based Predictions of Secondary and Coiled-Coil Structure

Sequence-based secondary structure predictions were performed using all of the prediction programs (SOPM, SOPMA, HNN, MLRC, DPM, DSC, GOR I, GOR III, GOR IV, PHD, PREDATOR, and SIMPA96) available on the NPS@web server [29]. The predictions of coiled-coil regions were done by the COILS prediction server [30] using the recommended window of 28 residues, MTK matrix (derived from the sequences of myosins, tropomyosins, and keratins). Since coiled-coils are often fibrous, solvent-exposed structures, all residues except for the internal a and d have a high probability of being hydrophilic. Giving equal weights to all positions therefore means being biased towards hydrophilic, charge-rich sequences. To avoid highly charged false positives a 2.5-fold weighting of positions a, d was used.

### 2.6. Sequence-Based Predictions of Disorder Propensity

Sequence-based predictions of the dynamic properties of individual proteins were obtained from the Predictors of Natural Disordered Regions (PONDR) prediction server [31]. The analysis of the disorder propensities of the MCP signal family was carried out using RONN disorder prediction software [32, 33].

## 3. Results

### 3.1. pHtrII-cyt has Strong Propensity for Helix

**Figure 1:**
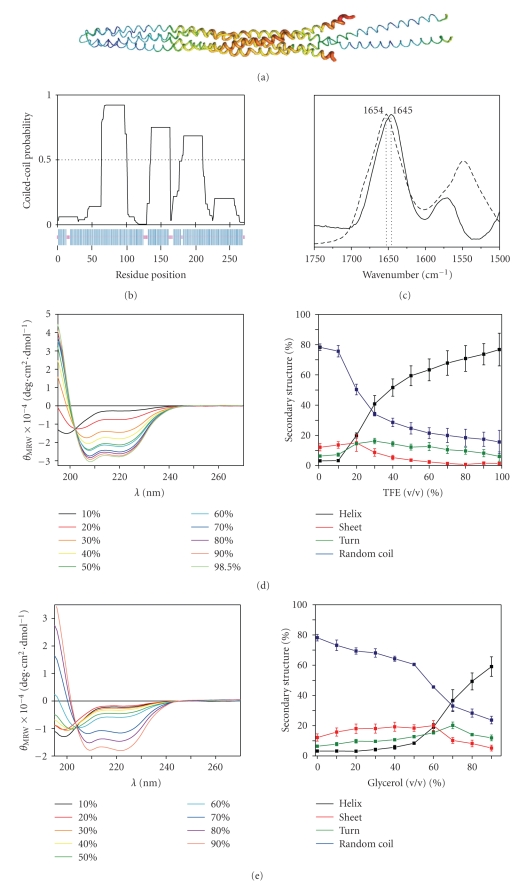

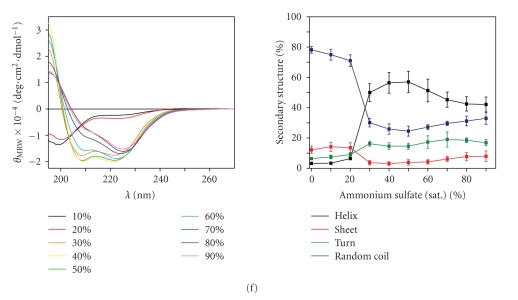
(a) X-ray crystal structure model of the Tsr-cyt (PDB ID 1QU7) corresponding to identical positions of the pHtrII-cyt investigated in this study. (b) Top, prediction of coiled-coil structure for pHtrII-cyt by the COILS software (Section 2.5) and below, prediction of secondary structure for pHtrII-cyt by the DSC software (Section 2.5). (c) Comparison of FTIR spectra of 10 mg/mL pHtrII-cyt in buffer C (solid line) and in dry film (dashed line) at room temperature. (d)–(f) Effects of increasing (d) TFE, (e) glycerol (v/v), and (f) ammonium sulfate (in % of saturation) concentrations on far-UV CD spectra of pHtrII-cyt recorded at protein concentration of 0.1 mg/mL at +20°C (left panel, colors correspond to TFE, glycerol, or ammonium sulfate concentration) and deduced secondary structure content (right panel, colors correspond to the content of secondary structure elements). The secondary structure content estimations were generated by the CONTIN/LL program from the CDPro package (Section 2.5); secondary structure elements are designated as follows: helix—black, sheet—red, turn—green, and random coil—blue.

pHtrII-cyt is predicted to be a helical coiled-coil based on homology to the serine receptor (Figure 1(a)), high coiled-coil structure propensity (Figure 1(b), top panel), and high helix propensity with 63% to 97% *α*-helix content (Figure 1(b), bottom panel), depending on the prediction algorithm used (see Section 2.5). These predictions are not consistent with the CD spectra recorded in the commonly used buffers, PBS, buffer A and buffer B, which were all indistinguishable and essentially lacked negative ellipticity at 222 nm characteristic for helix [20]. The values obtained from deconvolution of the spectra were ∼77% unstructured, ∼7% turn, ∼13% *β*-sheet, and ∼3% *α*-helix. However, control experiments with helix-inducing solvent 2,2,2-trifluoroethanol (TFE) showed a gradual rise in negative ellipticity at 222 nm with increasing amounts of TFE added (Figure 1(d)), experimentally validating the high helix propensity inferred from the pHtrII-cyt sequence.

### 3.2. Dehydration Stabilizes Helix Structure

Figure 1(c) shows FTIR spectra in the amide I region for pHtrII-cyt in buffer C (solid line) and in dry film (dashed line). The position of the maximum of the amide I band in buffer C at 1645 cm^−1^ is characteristic of a disordered conformation. In dry film, a shift to 1654 cm^−1^ was observed, a value that is characteristic of *α*-helical secondary structure [25, 34].

### 3.3. Small Molecule Osmolytes Stabilize Helix Structure

Addition of glycerol to PBS at concentrations of 60% (v/v) and higher greatly stabilized helix content which based on spectral deconvolution rose from ∼19% in 60% (v/v) glycerol to ∼59% in 90% (v/v) glycerol. *β*-sheet and turn contributed, respectively, ∼5% and ∼12% to the spectrum in 90% (v/v) glycerol (Figure 1(e)). In sucrose, at 90% (w/v) saturation, *α*-helix content was ∼37%, where *β*-sheet and turn contributed, respectively, ∼10% and ∼21%, to the spectrum (data not shown).

### 3.4. Salts Affect pHtrII-cyt Differentially

**Figure 2:**
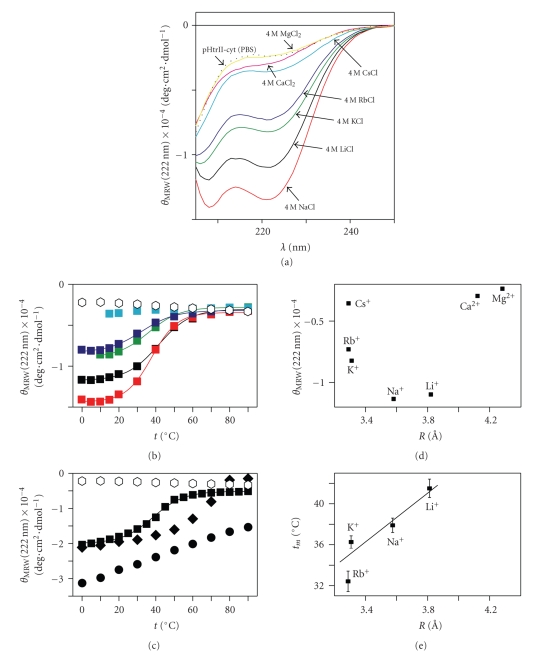
(a) Effect of addition mono- and bivalent salts on far-UV CD spectra of pHtrII-cyt recorded at protein concentration of 0.1 mg/mL at +20°C. Dotted line is the spectrum of pHtrII-cyt in PBS, black spectrum corresponds to the addition of 4 M LiCl, red—4 M NaCl, green—4 M KCl, blue—4 M RbCl, cyan—4 M CsCl, magenta—4 M CaCl_2_, and yellow—4 M MgCl_2_. (b) Thermal denaturation plots of pHtrII-cyt monitored at 222 nm in PBS (∘) and in PBS + monovalent salts (□). Colors are the same as in (a). (c) Thermal denaturation plots of pHtrII-cyt monitored at 222 nm in PBS (∘), PBS + 90% (v/v) glycerol (▪), PBS + 98.5% (v/v) TFE (•), and in PBS + 40% saturation ammonium sulfate (◆). The straight lines represent sigmoidal fits of the experimental data. Dependences of the mean residue molar ellipticity at 222 nm (d) and midpoints of thermal denaturation monitored at 222 nm (e) of pHtrII-cyt in PBS + 4 M of the corresponding chloride salt on the hydration radii of alkali cations used in the study. The straight line in (e) represents linear fit with correlation coefficient of 0.9. Note that the spectra in the presence of MgCl_2_ or CaCl_2_ were recorded in Tris buffer (buffer B, pH 7.2) instead of PBS because of the low solubility of these salts in PBS.

Ammonium sulfate, a common agent to precipitate, stabilize, or facilitate refolding of proteins, up to 30% saturation, did not significantly alter the CD spectra of pHtrII-cyt obtained in PBS alone (Figure 1(f)). Between 30% and 60%, pHtrII-cyt adopted approximately 50–56% *α*-helical conformation, while at above 60% saturation, helical content was reduced and that of random coil increased. Previous crosslinking and analytical gel-filtration experiments showed that the structural transition was accompanied by protein aggregation at high ammonium sulfate concentrations [20]. Since the native intracellular environment for *N. pharaonis* includes 4 M KCl, we also investigated the effect of monovalent and bivalent cationic solutions on pHtrII-cyt CD spectra. In solutions of up to 2 M of either LiCl, NaCl, KCl, RbCl, or CsCl in PBS and CaCl_2_ and MgCl_2_ in buffer B, pHtrII-cyt CD spectra exhibited predominantly unfolded features. However, at 4 M salt concentrations, helix conformation was stabilized to different degrees depending on the nature of the salt added (Figure 2(a)). A plot of hydrated ion size (ion radii taken from [35]) versus ellipticity (Figure 2(d)) shows a minimum at the position of NaCl, indicating that NaCl is best in stabilizing pHtrII-cyt structure. We note that maximum helix induction by the high salt additions required buffer D (containing phosphate and other salts) as the base buffer, suggesting that there is additional complexity in the factors contributing to maximum stability than can be explained by a single solvent component. This also reflects the complex solvent environment of the halophilic cytoplasm. For example, studies in *H. marismortui* indicated that although KCl was the predominant salt, there were also considerable amounts of sodium and magnesium salts present [36].

### 3.5. pHtrII-cyt Structure is Thermally Labile

CD was used to monitor thermal stability of pHtrII-cyt. The negative ellipticity at 222 nm as a function of temperature is shown in Figures 2(b) and 2(c). In PBS, CD spectra were essentially independent of temperature, characteristic of random coil at all temperatures probed (0°C to +99°C). In the presence of 4 M salt (Figure 2(b)), helicity was lost cooperatively with rising temperature with a single transition point, observed at +41.5°C in 4 M LiCl, +37.9°C in 4 M NaCl, +36.3°C in 4 M KCl, and +32.4°C in 4 M RbCl. The melting temperature was thus linearly dependent on ionic radius of the cations, with smaller ions stabilizing helix stronger (Figure 2(e)).

Denaturation in 90% (v/v) glycerol proceeded similar to that observed in the presence of high salt concentrations, that is, cooperatively with a transition point of +38.3°C. Thermal denaturation of pHtrII-cyt in PBS + 40% saturation ammonium sulfate was fitted by a sigmoidal curve with a transition point significantly higher than those obtained under any other condition, at +66 ± 3°C. However, the shape of the curve was not symmetric indicating the presence of multiple species with different melting temperatures. In 98.5% (v/v) TFE, a transition point of +36 ± 4.5°C was obtained, but seemed to follow a linear dependency with temperature, suggesting a gradual melting with little cooperativity.

### 3.6. NMR Spectroscopy Reveals High Dynamics of pHtrII-cyt

**Figure 3:**
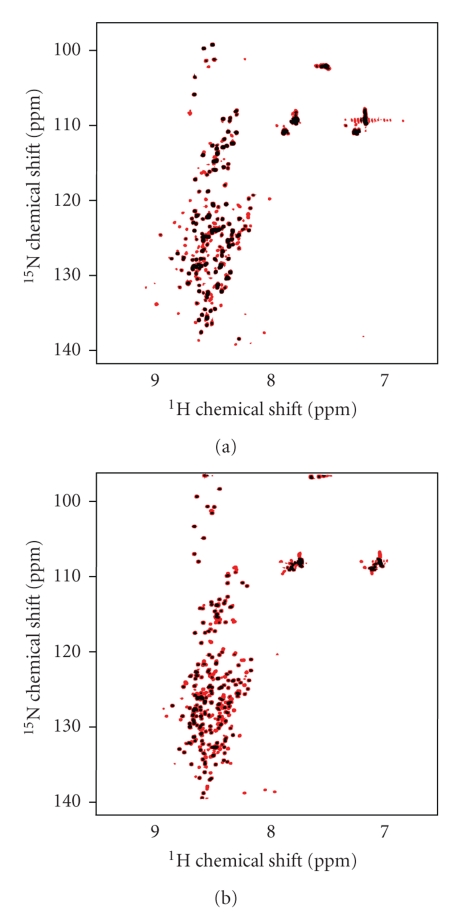
(a) 600 MHz ^1^H-^15^N HSQC spectrum of 5 mg/mL pHtrII-cyt in buffer D plus 4 M KCl at +10°C (red) and +20°C (black) recorded in thin high salt tubes. Signals correspond to backbone and side-chain NH or NH_2_ groups. There is no Trp in the pHtrII-cyt sequence, so there are no resonances expected beyond 10 ppm. (b) Same as in (a) measured in buffer D using the same type of NMR tubes.

Resonances in one-dimensional ^1^H spectra of pHtrII-cyt in buffer D (data not shown) were very sharp and clustered around 8.3 ppm. No resonances were observed upfield of 0.8 ppm. These spectral features are characteristic of disordered or globally dynamic proteins [37]. ^1^H-^15^N-HSQC spectra of uniformly ^15^N-labeled pHtrII-cyt at +10°C and +20°C in buffer D with and without 4 M KCl are shown in Figures 3(a), 3(b), respectively. Spectra recorded at +4°C, +10°C, +25°C, +37°C, and +41°C were qualitatively similar in chemical shift dispersion but varied greatly with respect to individual chemical shifts (data not shown). A maximum of 254 of the expected 271 backbone NH resonances were resolved at +4°C in buffer D, while only 216 were resolved at +41°C in buffer D. Only 27 peak positions were independent of temperature for low salt and 44 peak positions for high salt samples. To quantify the dynamic properties of HtrII-cyt, we measured ^15^N relaxation parameters *T*_1_, *T*_2_, and hetNOE at two different temperatures (+10°C and +20°C) in the presence and absence of 4 M KCl. The results are listed in Table 1. Values could only be obtained for 141–148 of the 251–258 peaks observed in the four respective HSQC spectra, while 106–110 of the peaks decayed so rapidly that reliable relaxation parameters could not be determined. This indicates that the backbone resonances of these residues are most likely rigid or undergo conformational exchange on the microsecond-to-millisecond time scale. For the 141–148 peaks for which relaxation parameters were determined in buffer D at +10°C and +20°C, the *T*_1_, *T*_2_ and hetNOE values are characteristic of random coil structure as, for example, observed in unfolded lysozyme in 8 M urea [38]. In contrast, the observed *T*_1_, *T*_2_, and hetNOE values are far from those observed in a rigid helical protein. There was very little deviation from the average in all relaxation parameters at both temperatures suggesting that the subset of resonances for which relaxation parameters could be measured experiences similarly restricted motions, on the nanosecond-to-subnanosecond time scale. In the presence of 4 M KCl, hetNOE values were significantly less negative at both temperatures. *T*_2_ average values were overall slightly increased as compared to those observed in the absence of high salt, probably due to the difference in solvent viscosity. Importantly, however, the standard deviation in *T*_2_ times was much higher in the presence of salt, reflecting the appearance of resonances with lower *T*_2_ times (higher relaxation rates), indicating restriction in conformational space similar to what has been observed in hydrophobic clusters in unfolded lysozyme [38].

These results strongly suggest that both in the presence and absence of salt, there is significant restriction in the conformational space being explored by the ensemble of structures. The NMR results provide detailed information on the number of resonances whose dynamics are affected by changes in solvent conditions. For 141–148 backbone amide groups out of 271 pHtrII-cyt residues (52–55%), relaxation rates indicated random-coil behavior under low salt conditions. These represent the most flexible regions of pHtrII-cyt. In these regions, the presence of 4 M KCl diminishes the degree of flexibility significantly. In the remaining 45–48% of resonances, there is stronger restriction in conformational space that is present both in low and high salt conditions. These residues experience significant changes in their environment when comparing low and high salt conditions because the chemical shift values change and a broader range of values is found under high salt conditions indicating more highly structured states.

### 3.7. Disorder Is Predicted to be a Conserved Feature of the MCP Family

**Figure 4:**
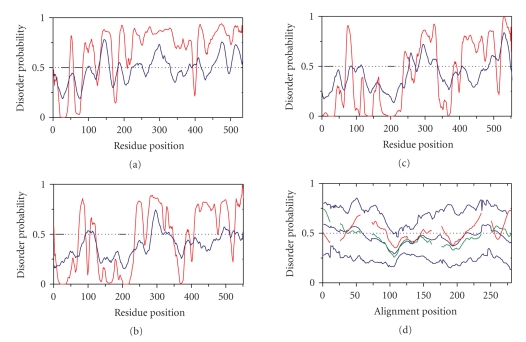
PONDR (red lines) and RONN (blue lines) predictions of disorder with transmembrane segments designated by black bars for (a) pHtrII (Swiss-Prot ID P42259), (b) Tsr (Swiss-Prot ID P02942), (c) Tar (Swiss-Prot ID P07017), (d) disorder propensities for MCP family members estimated using RONN. Mean, maximum, minimum calculated for each position in the alignment are shown as thin blue lines. The corresponding values for the pHtrII-cyt and the Tsr-cyt are shown as thick red and green lines, respectively.

The experimental evidence presented above uniformly suggests that the pHtrII-cyt structure is highly dynamic. Since the Tar-cyt and Tsr-cyt structures are also believed to be dynamic (see Section 1), we investigated the propensity for disorder in all three proteins and in the entire MCP family using two methods: PONDR [31] and RONN [32, 33] (Figure 4). *Y*-axis values (probability of disorder) higher than 0.5 generally indicate disordered regions. In all three proteins PONDR predicts substantial disorder for the cytoplasmic domain, while overall the RONN probabilities for disorder were more moderate (Figure 4(a)). Using RONN, we investigated the propensity for disorder in the entire MCP family (Figure 4(d)). A mean value of 0.5 with a low standard deviation of ~0.09 was obtained. Tsr-cyt and pHtrII-cyt closely follow the mean RONN disorder values in the MCP family at the majority of sites, indicating that high flexibility but not complete lack of structure is conserved in the MCP family. Notably, some regions at cytoplasmic domain alignment position 100–110, 190–200 and at the extreme C-terminus (>275) displayed least flexibility in the MCP family (Figure 4(d)). These correspond to regions of lowest B-factors observed in the Tsr-cyt crystal structure [13].

## 4. Discussion

### 4.1. A Model for pHtrII-cyt Structure

Previous studies showed that the shape of pHtrII-cyt is an elongated rod with a radius of gyration of 54 Å [20], both as a monomer in low salt and as a dimer in 4 M KCl. These high KCl concentrations are physiological for *N. pharaonis* [39, 40]. This shape is consistent with the helical coiled-coil dimer of the Tsr-cyt crystal structure [13].

However, this shape is not consistent with CD data lacking evidence for helix altogether for the monomer, and indicating only 24% helix for the dimer in 4 M KCl [20]. Here, we provide evidence that despite “random coil” spectroscopic features, pHtrII-cyt can exist as a highly flexible, loosely packed but folded helical coiled-coil: (i) sequence-based structure predictions all strongly support a helical coiled-coil structure for pHtrII-cyt, and addition of TFE, a well-known helix inducing agent, is able to generate CD spectra that are characteristic for ~80% helix; (ii) both, uncharged electrolytes and salts induce negative ellipticity in CD spectra of pHtrII-cyt, but the thermal midpoint transitions occur at very low temperatures indicating thermal instability; (iii) the majority of signals in ^1^H, ^15^N-HSQC spectra are highly temperature-sensitive, with the largest number of visible peaks observed at low temperatures and best spectral dispersion in high salt at low temperatures. Because small angle neutron scattering indicates an identical radius of gyration [20] regardless of the differences in helix content in CD spectra and spectral changes in the NMR spectra, we propose that stabilization of more folded conformations by added stabilizing agents in the case of pHtrII-cyt means a reduction in dynamics leading to a more compact but overall similar structure.

**Figure 5:**
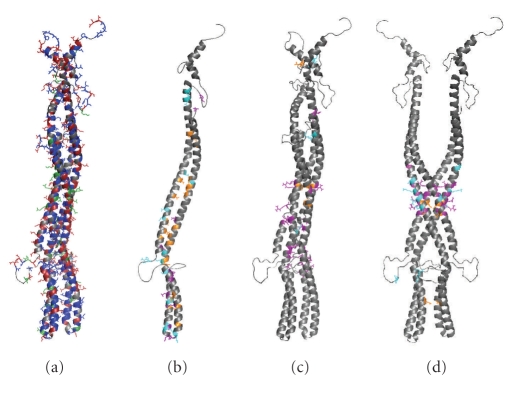
(a) Homology model of pHtrII-cyt based on the structure of Tsr-cyt. Negatively charged amino acids are shown in red, positively charged amino acids in blue, and hydrophobic amino acids in green. (b) FastContact analysis of each helix in a coiled-coil monomer chain. Amino acids contributing to favorable electrostatic interactions are shown in cyan, those contributing to free energy contacts in orange, and those contributing to both are shown in purple. (c) FastContact analysis of the dimer interface. Colors as in (b), (d). Docking of the two monomeric chains using ClusPro software. Colors from FastContact analysis as in (b), (c).

The stabilization of folded structures of proteins by additives has been extensively studied beginning with the seminal work by Hofmeister [41]. Careful experiments, thermodynamic analyses, and discussions over the years ([42–45] and references within), including those for halophilic proteins [46–49], have led to the relatively recent “unifying theory” that both, “hard” factors, referring to excluded volume effects and “soft” factors, referring to interactions between additives, water, and proteins/nucleic acids together contribute to the mechanisms underlying the effects of cosolvents on protein/nucleic acid structural stability. While it is possible to empirically estimate the overall contribution of hard versus soft effects [44], the current uncertainty of the nature of unfolded states will preclude quantitative modeling in the near future [50]. The very high density of acidic amino acids in pHtrII-cyt typical of halophilic proteins suggests that “soft” effects will be important in the case of stabilization of the helical coiled-coil in pHtrII-cyt. To illustrate this point, we generated homology models of each monomer in the dimer from the Tsr-cyt crystal structure (Figure 5(a)). FastContact analysis [51] of the model reveals that this dimeric structure is not stable because of electrostatic repulsion between the acidic residues at the dimer interface (Figure 5(c)), while each helix in the monomer is favorably packed through hydrophobic interactions (Figure 5(b)). As a result, docking of the two helical monomers using ClusPro software [52] does not reproduce the homology model, and instead, only results in physical interaction in an area in which hydrophobic interactions are possible (Figure 5(d)). Presumably, the high salt concentrations in vivo overcome the electrostatic repulsions. To test this hypothesis, we conducted systematic comparison of monovalent and bivalent cations in the presence of a constant anion (chloride). We found that the extent and stability of the ellipticity signal in CD spectra in the presence of different cations follows exactly that predicted by Collins' law of matching water affinities [53]: the maximum stabilization is with the ion (Na^+^) that has a water affinity closest to those of the acidic amino acid residues in pHtrII-cyt, and not the one with highest charge density (Ca^2+^), or smallest ionic radius (Li^+^). However, even in the presence of 4 M NaCl, pHtrII-cyt is still a dynamic ensemble of folded coiled-coil structures, rather than a single stable conformation.

### 4.2. Why is pHtrII-cyt so Dynamic?

The dynamic properties of pHtrII-cyt reported here for high salt concentrations physiological for *N. pharaonis* are in good agreement with those reported for Tar-cyt under conditions physiological for *E. coli* [16]. Furthermore, Tar-cyt and Tsr-cyt were found to be globally dynamic in solution [18], similar to our observations with pHtrII-cyt. Finally, disorder predictions indicate conservation of flexible properties in the entire MCP family. This evolutionary conservation of flexibility suggests a role in function. Activation of chemotaxis receptors and phototaxis transducers occurs through very subtle effects such as small helix movements during activation and only dimers bind and activate CheA [54–56]. The compaction coupled to dimerization of a preformed ensemble of coiled-coil helical-like structures proposed here could contribute to the mechanism for activation. In support of this hypothesis, the strength of the interaction between helices in the chemoreceptor cytoplasmic domain was found to modulate kinase activity [54, 55, 57].

### 4.3. Implications for Study of “Intrinsically Unstructured” Proteins

Recently, it was estimated that as much as 50% of human proteins belong to the “intrinsically unstructured” or “natively unfolded” class [58]. These estimates are based on predictors of disorder such as PONDR that are trained in part on sequence data of proteins with CD, FTIR, or NMR spectra like the ones we measured for pHtrII-cyt as sole evidence for disorder [59]. The findings presented in this paper supporting that pHtrII-cyt is not a random coil suggest that the terms “intrinsically unstructured” and “natively unfolded” would more appropriately be termed “highly dynamic structural ensemble." The notion of residual structure is analogous to that observed in chemically denatured proteins, where it has been proposed that a true random coil may never exist [60]. The case of pHtrII-cyt is particularly intriguing because its nonspherical shape allows us to clearly distinguish between a true random coil that may or may not retain residual structure and a near-native but highly dynamic structural ensemble. This case also reminds us that local, that is, secondary structure in the classical sense and long-range tertiary structure defining overall shape are not independent of each other. Understanding the amplitude of the motions observed in this and other systems would be an important future goal towards defining this interrelationship in more detail. Investigating how much motion is needed to lose the characteristic ellipticipty in CD spectra would be a good starting point [26].

### 4.4. Relation to in Vivo Structure and Dynamics

It is an unanswered question to what extent proteins experimentally observed to be dynamic in vitro are as dynamic in vivo [42, 61]. Evidence suggests that crowding inside the cell can be mimicked with solutes in vitro. For example, FlgM, an intrinsically disordered protein gains structure in the presence of high concentrations of solutes in vitro and inside living *E. coli* cells [62]. Low stability of intrinsically unstructured or highly dynamic protein ensembles has been shown to be related to functions such as membrane insertion or fusion events, protein interactions, and signal transduction [63].

## Figures and Tables

**Table 1:** Mean and standard deviation of *T*_1_, *T*_2_, and hetNOE values measured at different temperatures in buffer D in the presence and absence of 4 M KCl on a 600 MHz instrument.

Temperature and solvent	*T*_1_(s)	*T*_2_(s)	hetNOE
+10°C-buffer D	0.67 ± 0.18	0.28 ± 0.11	−0.22 ± 0.54
+10°C-buffer D plus 4 M KCl	0.66 ± 0.22	0.33 ± 0.28	−0.08 ± 0.30
+20°C-buffer D	0.74 ± 0.34	0.31 ± 0.15	−0.35 ± 0.57
+20°C-buffer D plus 4 M KCl	0.65 ± 0.24	0.38 ± 0.43	−0.12 ± 0.27
